# Supplementary material for: Genome Size in the Arenaria ciliata Species Complex (Caryophyllaceae), with Special Focus on A. ciliata subsp. bernensis, a Narrow Endemic of the Swiss Northern Alps
Source: Plants (Basel). 2022 Dec 13;11(24):3489. doi: 10.3390/plants11243489 (PMC9783149; doi:10.3390/plants11243489)
Supplement: Supplementary file 1 [file plants-11-03489-s001.zip › plants-2094521-supplementary.pdf]

Supplementary Materials

Table S1. Characterization of all collected taxa and samples from the *Arenaria ciliata* species complex, with the corresponding genome sizes (2c values in pg of DNA). Irregular floral morphology: * plants with 6 petals, ** plants with 9 petals. x- and y- coordinates indicate the National Swiss Geographic Mapping reference.

Taxon	Date	x-Coordinates	y-Coordinates	Region / Summit	Elevation (m a.s.l.)	2c (pg)
A. ciliata subsp. *bernensis*	19.08.2022	2567765	1155653	Moléson	1920	6.74
A. ciliata subsp. *bernensis*	19.08.2022	2567765	1155653	Moléson	1920	6.62
A. ciliata subsp. *bernensis*	19.08.2022	2567744	1155684	Moléson	1840	6.34
A. ciliata subsp. *bernensis*	19.08.2022	2567744	1155684	Moléson	1840	6.26
A. ciliata subsp. *bernensis*	19.08.2022	2567744	1155684	Moléson	1840	6.53
A. ciliata subsp. *bernensis*	05.08.2022	2577300	1152381	Vanil Noir / Vanil de l'Ecri	2365	6.42
A. ciliata subsp. *bernensis*	30.08.2022	2578797	1154815	Vanil Noir / Galère	2100	6.40
A. ciliata subsp. *bernensis*	30.08.2022	2578495	1154467	Vanil Noir / Galère	2100	6.65
A. ciliata subsp. *bernensis*	30.08.2022	2577920	1154018	Vanil Noir / Galère	2100	6.74
A. ciliata subsp. *bernensis*	30.08.2022	2578859	1154868	Vanil Noir / Galère	2100	6.99
A. ciliata subsp. *bernensis*	30.08.2022	2578893	1154981	Vanil Noir / Galère	2100	6.65
A. ciliata subsp. *bernensis*	30.08.2022	2578163	1154140	Vanil Noir / Galère	2100	6.45
A. ciliata subsp. *bernensis*	30.08.2022	2578091	1154111	Vanil Noir / Galère	2100	6.89
A. ciliata subsp. *bernensis*	05.08.2022	2579688	1155723	Vanil Noir / Dent de Brenleire	2100	7.33
A. ciliata subsp. *bernensis*	17.08.2022	2579688	1155723	Vanil Noir / Dent de Brenleire	2300	6.56
A. ciliata subsp. *bernensis*	17.08.2022	2579680	1155729	Vanil Noir / Dent de Brenleire	2300	6.77
A. ciliata subsp. *bernensis*	17.08.2022	2579731	1155635	Vanil Noir / Dent de Brenleire	2300	6.80
A. ciliata subsp. *bernensis*	17.08.2022	2579675	1155733	Vanil Noir / Dent de Brenleire	2300	7.02
A. ciliata subsp. *bernensis*	15.08.2022	2580095	1154148	Vanil Noir / Dzori Marro	1800	7.16
A. ciliata subsp. *bernensis*	21.08.2022	2585594	1163489	Schopfenspitz	2090	6.89
A. ciliata subsp. *bernensis*	21.08.2022	2585594	1163489	Schopfenspitz	2090	7.09
A. ciliata subsp. *bernensis*	21.08.2022	2585594	1163489	Schopfenspitz	2090	7.02
A. ciliata subsp. *bernensis*	21.08.2022	2585573	1163458	Schopfenspitz	2080	6.99
A. ciliata subsp. *bernensis*	21.08.2022	2585573	1163458	Schopfenspitz	2080	6.96
A. ciliata subsp. *bernensis*	28.08.2022	2590513	1166413	Kaiseregg / Kaisereggpass	2090	6.93
A. ciliata subsp. *bernensis*	28.08.2022	2590513	1166413	Kaiseregg / Kaisereggpass	2090	6.80
A. ciliata subsp. *bernensis*	28.08.2022	2590513	1166413	Kaiseregg / Kaisereggpass	2090	6.83
A. ciliata subsp. *bernensis*	28.08.2022	2590535	1166783	Kaiseregg / Kaisereggpass	2070	6.93
A. ciliata subsp. *bernensis*	28.08.2022	2590769	1166752	Kaiseregg	2170	6.56
A. ciliata subsp. *bernensis*	28.08.2022	2590769	1166752	Kaiseregg	2170	6.45
A. ciliata subsp. *bernensis*	28.08.2022	2590769	1166752	Kaiseregg	2170	6.65
A. ciliata subsp. *bernensis*	28.08.2022	2590769	1166752	Kaiseregg	2170	6.71
A. ciliata subsp. *bernensis*	28.08.2022	2590769	1166752	Kaiseregg	2170	6.74
A. ciliata subsp. *bernensis*	05.08.2022	2601038	1172674	Gantrisch	2110	7.19
A. ciliata subsp. *bernensis*	05.08.2022	2601038	1172674	Gantrisch	2110	6.71
A. ciliata subsp. *bernensis*	05.08.2022	2601038	1172674	Gantrisch	2110	6.89
A. ciliata subsp. *bernensis*	05.08.2022	2601102	1172674	Gantrisch	2140	7.16
A. ciliata subsp. *bernensis*	05.08.2022	2601102	1172674	Gantrisch	2140	7.19
A. ciliata subsp. *bernensis***	05.08.2022	2601102	1172674	Gantrisch	2140	6.83
A. ciliata subsp. *bernensis*	05.08.2022	2601400	1172581	Gantrisch / Leiterenpass	1940	6.88
A. ciliata subsp. *bernensis*	05.08.2022	2601400	1172581	Gantrisch / Leiterenpass	1940	6.96
A. ciliata subsp. *bernensis*	05.08.2022	2601400	1172581	Gantrisch / Leiterenpass	1940	7.09
A. ciliata subsp. *bernensis*	05.08.2022	2601400	1172581	Gantrisch / Leiterenpass	1940	6.80
A. ciliata subsp. *bernensis*	05.08.2022	2601400	1172581	Gantrisch / Leiterenpass	1940	7.16
A. ciliata subsp. *bernensis*	05.08.2022	2601400	1172581	Gantrisch / Leiterenpass	1940	6.99
A. ciliata subsp. *bernensis*	05.08.2022	2601400	1172581	Gantrisch / Leiterenpass	1940	6.80

A. ciliata subsp. *bernensis*	05.08.2022	2601400	1172581	Gantrisch / Leiterenpass	1940	7.09
A. ciliata subsp. *bernensis*	05.08.2022	2601400	1172581	Gantrisch / Leiterenpass	1940	7.26
A. ciliata subsp. *bernensis*	05.08.2022	2601400	1172581	Gantrisch / Leiterenpass	1940	7.09
A. ciliata subsp. *bernensis*	12.08.2022	2607498	1171412	Stockhorn	2110	7.75
A. ciliata subsp. *bernensis*	12.08.2022	2607498	1171412	Stockhorn	2110	7.09
A. ciliata subsp. *bernensis*	12.08.2022	2607498	1171412	Stockhorn	2110	7.48
A. ciliata subsp. *bernensis*	12.08.2022	2607498	1171412	Stockhorn	2110	6.99
A. ciliata subsp. *bernensis*	12.08.2022	2607498	1171412	Stockhorn	2110	7.78
A. ciliata subsp. *bernensis**	12.08.2022	2607498	1171412	Stockhorn	2110	7.75
A. ciliata subsp. *bernensis**	12.08.2022	2607498	1171412	Stockhorn	2110	6.80
A. ciliata subsp. *bernensis**	12.08.2022	2607498	1171412	Stockhorn	2110	7.37
A. ciliata subsp. *ciliata*	05.08.2022	2567646	1155404	Moléson	1980	1.80
A. ciliata subsp. *ciliata*	05.08.2022	2567646	1155404	Moléson	1980	1.70
A. ciliata subsp. *ciliata*	05.08.2022	2567646	1155404	Moléson	1980	1.65
A. ciliata subsp. *ciliata*	10.08.2022	2576024	1150337	Vanil Noir / Gros Perré	2150	1.69
A. ciliata subsp. *ciliata*	10.08.2022	2576024	1150337	Vanil Noir / Gros Perré	2150	1.69
A. gothica	01.10.2022	2510831	1165911	Lac de Joux (ex situ BGFR)	1004	3.60
A. gothica	01.10.2022	2510831	1165911	Lac de Joux (ex situ BGFR)	1004	3.79
A. gothica	01.10.2022	2510831	1165911	Lac de Joux (ex situ BGFR)	1004	3.74
A. gothica	01.10.2022	2510831	1165911	Lac de Joux (ex situ BGFR)	1004	3.62
A. gothica	01.10.2022	2510831	1165911	Lac de Joux (ex situ BGFR)	1004	3.75
A. gothica	01.10.2022	2510831	1165911	Lac de Joux (ex situ BGFR)	1004	3.77
A. gothica	01.10.2022	2510831	1165911	Lac de Joux (ex situ BGFR)	1004	3.63
A. gothica	01.10.2022	2510831	1165911	Lac de Joux (ex situ BGFR)	1004	3.74
A. gothica	01.10.2022	2510831	1165911	Lac de Joux (ex situ BGFR)	1004	3.60
A. gothica	01.10.2022	2510831	1165911	Lac de Joux (ex situ BGFR)	1004	3.66
A. multicaulis	30.08.2022	2578601	1154553	Vanil Noir / Galère	2100	1.53
A. multicaulis	05.08.2022	2601038	1172519	Gantrisch	2020	1.60
A. multicaulis	05.08.2022	2601038	1172519	Gantrisch	2020	1.58
A. multicaulis	05.08.2022	2601038	1172519	Gantrisch	2020	1.63
A. multicaulis	05.08.2022	2601038	1172519	Gantrisch	2020	1.53

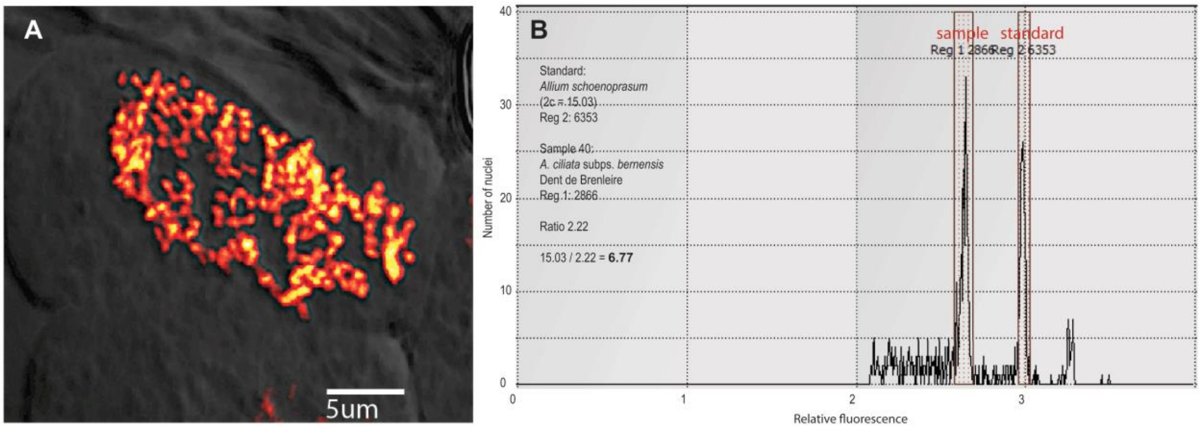

Figure S1. A – Confocal micrograph image of Fuelgen-stained late metaphase chromosomes in *Arenaria ciliata* subsp. *bernensis* from Dent de Brenleire (2n = 200, Fribourg, Switzerland). Image scale 1000x. **B** – Genome size estimation for *A. ciliata* subsp. *bernensis* from Dent de Brenleire using flow cytometry (for more details see Materials and Methods).
